# Towards elimination of lymphatic filariasis in southeastern Madagascar: Successes and challenges for interrupting transmission

**DOI:** 10.1371/journal.pntd.0006780

**Published:** 2018-09-17

**Authors:** Andres Garchitorena, Estelle M. Raza-Fanomezanjanahary, Sedera A. Mioramalala, Cédric B. Chesnais, Claude A. Ratsimbasoa, Herinirina Ramarosata, Matthew H. Bonds, Holivololona Rabenantoandro

**Affiliations:** 1 UMR 224 MIVEGEC, Institut de Recherche pour le Développement, Montpellier, France; 2 PIVOT, Ifanadiana, Madagascar; 3 Institut National de la Sante Publique et Communautaire, – Ministère de la Santé Publique, Ministère de l’Enseignement Supérieur et de la Recherche Scientifique, Antananarivo, Madagascar; 4 Direction de lutte contre le paludisme, – Ministère de la Santé Publique, Antananarivo, Madagascar; 5 UMI 233, Institut de Recherche pour le Développement (IRD), Université Montpellier, INSERM Unité 1175, Montpellier, France; 6 Department of Global Health and Social Medicine, Harvard Medical School, Boston, Massachusetts, United States of America; 7 Service de Lutte contre les Maladies Epidémiques et Négligées – Ministère de la Santé Publique, Antananarivo, Madagascar; Washington University School of Medicine, UNITED STATES

## Abstract

**Introduction:**

A global strategy of mass drug administration (MDA) has greatly reduced the burden of lymphatic filariasis (LF) in endemic countries. In Madagascar, the National Programme to eliminate LF has scaled-up annual MDA of albendazole and diethylcarbamazine across the country in the last decade, but its impact on LF transmission has never been reported. The objective of this study was to evaluate progress towards LF elimination in southeastern Madagascar.

**Methods:**

Three different surveys were carried out in parallel in four health districts of the Vatovavy Fitovinany region in 2016: i) a school-based transmission assessment survey (TAS) in the districts of Manakara Atsimo, Mananjary, and Vohipeno (following a successful pre-TAS in 2013); ii) a district-representative community prevalence survey in Ifanadiana district; and iii) a community prevalence survey in sentinel and spot-check sites of these four districts. LF infection was assessed using the Alere Filariasis Test Strips, which detect circulating filarial antigens (CFA) of adult worms. A brief knowledge, attitudes and practices questionnaire was included in the community surveys.

**Principal findings:**

None of the 1,825 children sampled in the TAS, and only one in 1,306 children from sentinel and spot-check sites, tested positive to CFA. However, CFA prevalence rate in individuals older than 15 years was still high in two of these three districts, at 3.5 and 9.7% in Mananjary and Vohipeno, respectively. Overall CFA prevalence in sentinel and spot-check sites of these three districts was 2.80% (N = 2,707), but only two individuals had detectable levels of microfilaraemia (0.06%). Prevalence rate estimates for Ifanadiana were substantially higher in the district-representative survey (15.8%; N = 545) than in sentinel and spot-check sites (0.8%; N = 618). Only 51.2% of individuals surveyed in these four districts reported taking MDA in the last year, and 42.2% reported knowing about LF.

**Conclusions:**

Although TAS results suggest that MDA can be stopped in three districts of southeastern Madagascar, the adult population still presents high CFA prevalence levels. This discordance raises important questions about the TAS procedures and the interpretation of their results.

## Author summary

Lymphatic filariasis is a neglected disease with chronic disabling consequences. Endemic countries have reduced lymphatic filariasis transmission through a strategy of annual rounds of mass drug administration (MDA), but the impact of such strategy has not yet been reported for Madagascar. In this study we conducted three different surveys and used rapid diagnostic tests to evaluate lymphatic filariasis transmission in four health districts of southeastern Madagascar. This included a school-based transmission assessment survey (TAS), the international gold standard to help national programmes confirm that they have interrupted lymphatic filariasis transmission, and two complementary community-based surveys. Our TAS results suggested that MDA could be stopped in three districts, confirming the consistent decline in lymphatic filariasis observed in recent years. However, the other two surveys revealed that the adult population still had high prevalence levels. This discordance raises questions about the TAS procedures and the interpretation of their results in contexts where, like in Madagascar, implementation of MDA is different for school age children than for the rest of the population.

## Introduction

The world is on a path towards the elimination of lymphatic filariasis (LF), a major neglected tropical disease (NTD) and parasitic infection transmitted by mosquitoes and caused by *Wuchereria bancrofti*, *Brugia malayi*, and *B*. *timori*. LF can result in chronic disabling consequences (e.g. hydrocele, lymphoedema and elephantiasis), and is responsible for about 5.8 million Disability Adjusted Life Years (DALYs) [1], including a substantial burden on mental health [2]. The availability of cheap, safe and effective treatments against the filarial infection, with long-term effects on disease transmission, has driven this international effort and allowed for a strategy of mass drug administration (MDA) to populations where LF is endemic (a combination of albendazole plus ivermectin or diethylcarbamazine) [3]. Following the launch of the Global Programme to Eliminate Lymphatic Filariasis in 2000 to achieve elimination by 2020 [4], National Programmes, the cornerstones of this effort, have been set up in all endemic countries. By 2015 they had distributed more than 6 billion treatments, covering nearly 60% of the world population requiring MDA [5]. As a result, transmission of LF had been interrupted by 2014 in 18 out of 73 endemic countries, and many others had achieved partial elimination [5].

Lymphatic filariasis is endemic in Madagascar, where it has been known for more than a century [6]. Surveys conducted in the 1950s and 70s revealed stable prevalence levels of microfilaraemia in the eastern coast of the island at about 25% [7,8], higher than other regions of Madagascar [8]. A country-wide mapping study in 2004–2005, at baseline of the National Programme, revealed that LF was endemic in 98 health districts out of 114 and more than 18 million people required MDA [9]. Since then, annual rounds of albendazole and diethylcarbamazine have been administered in endemic districts to people of all ages, except for children under 2 years old and pregnant women. MDA was initiated in districts of the eastern coast and has been progressively scaled-up over the past 10 years, currently reaching 62 districts at different stages of MDA (3 to 10 rounds). As a result, the National Programme needed to evaluate whether transmission has been interrupted in these districts in order to decide on whether to continue with MDA.

WHO recommendations for the monitoring and evaluation of National Programmes involve surveillance of prevalence levels in sentinel and spot-check sites (individuals 5 years or older, including adults), followed by a confirmation that LF transmission has been interrupted through a transmission assessment survey (TAS) after certain criteria are met: a TAS is carried out in populations that have received MDA for at least 5 years (minimum coverage of 65%) and where prevalence of microfilaraemia, in sentinel and spot-check sites, has been reduced to less than 1% (or antigenaemia <2%) after the last effective round [10]. The survey is conducted in primary schools, targeting children between 6 and 7 years of age. In endemic areas for *W*. *bancrofti*, the surveys use point of care tests that detect circulating filarial antigens (CFA) of adult worms. In areas where *Anopheles* are the principal vectors, a proportion of positive CFA < 2% implies a recommendation for stopping MDA and beginning post-MDA surveillance [3]. Since 2013, these surveys use the Filariasis Test Strip (FTS) as the preferred CFA test, which has better sensitivity and stability than the previously used BinaxNOW Filariasis immunochromatographic card test (ICT; both produced by Alere, Scarborough, ME) [11,12].

**Fig 1:**
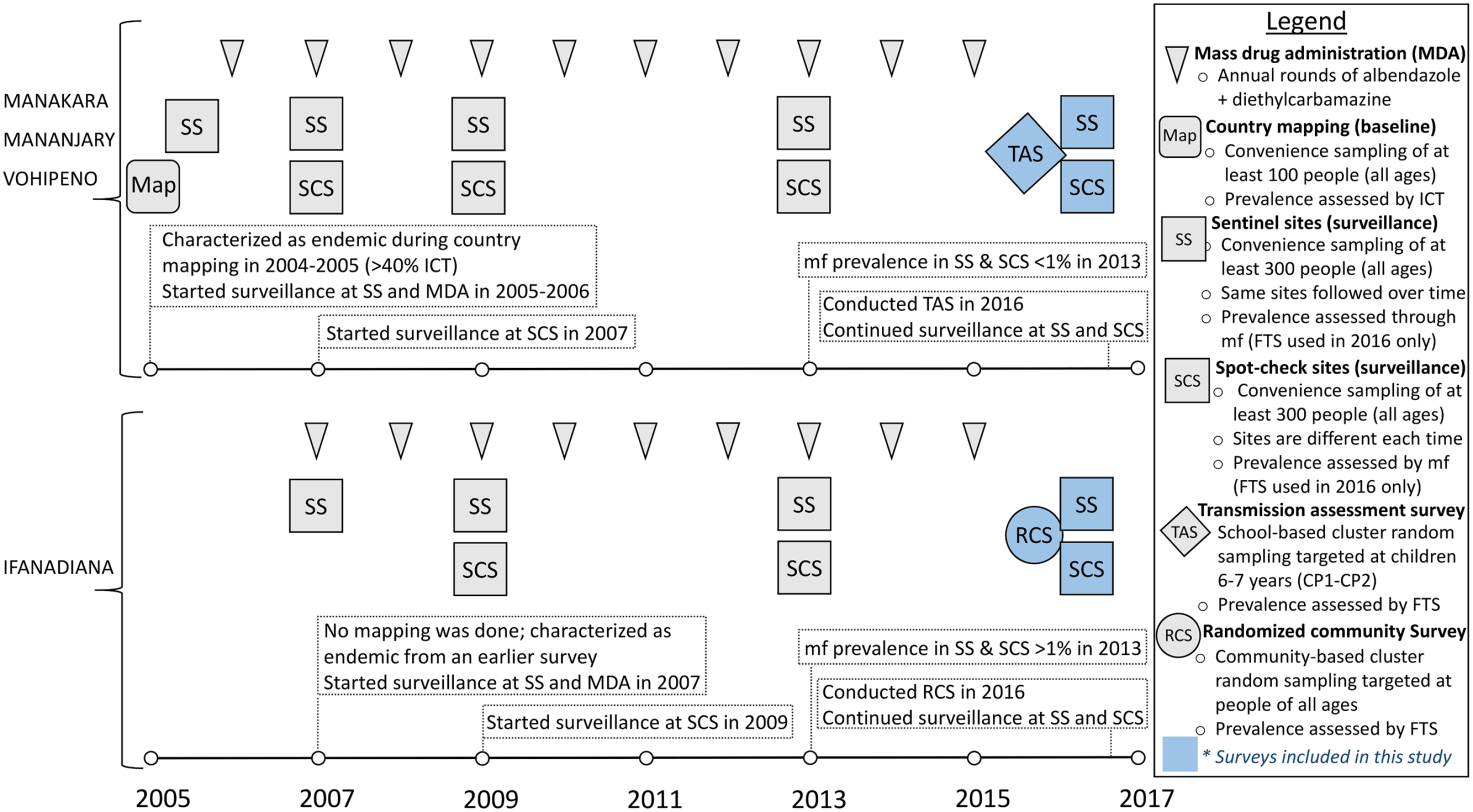
Chronology and main characteristics of mass drug administration, monitoring and evaluation implemented in the four health districts included in this study, Vatovavy Fitovinany region. **Source**: Madagascar Ministry of Health, National Programme for the Elimination of Lymphatic Filariasis.

The objective of this study was to evaluate LF transmission in four districts of the Vatovavy Fitovinany region (Eastern Madagascar) that had received 9–10 annual rounds of MDA, by comparing results obtained from three cross-sectional surveys, including a school-based TAS and two community-based prevalence surveys (Fig 1). All surveys took place in the fall of 2016 and used FTS to evaluate infection levels.

## Methods

### Study site and population

**Fig 2:**
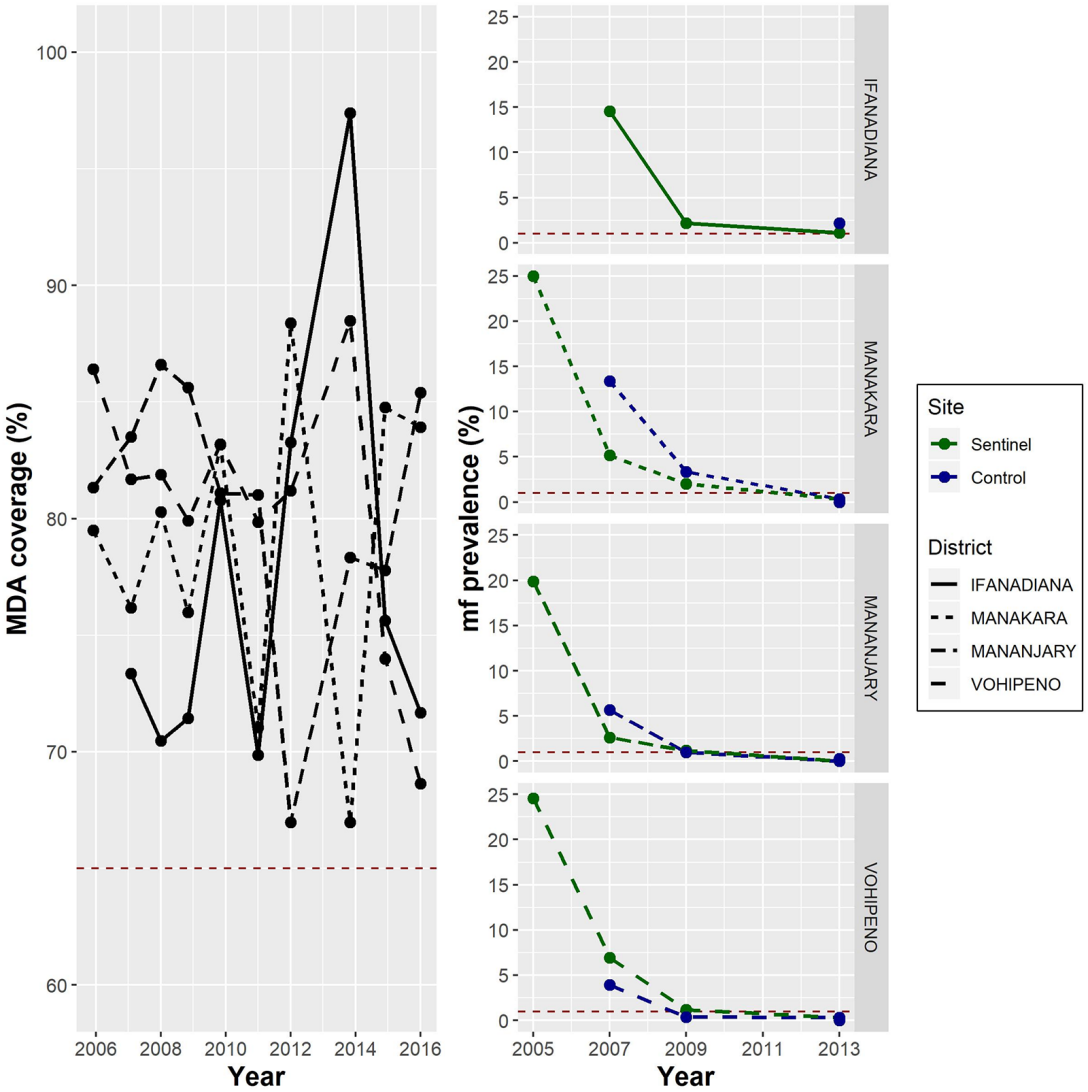
Evolution of lymphatic filariasis mf prevalence (right) and treatment coverage of mass drug administration (left) in four health districts of the region of Vatovavy Fitovinany, 2006–2016. Red dashed lines show threshold limits for eligibility to conduct a transmission assessment survey: 65% MDA coverage over 5 years and less than 1% mf prevalence in sentinel and spot check sites. **Source**: Madagascar Ministry of Health, National Programme for the Elimination of Lymphatic Filariasis. For mass drug administration (MDA) coverage estimations, uptake information in paper forms filled by schools and community health workers during MDA campaigns was aggregated by the head of the corresponding health centre, who calculated coverage using Ministry of Health population estimates for each administrative unit. These data were aggregated for each district by the National Programme. Selection of sentinel and spot-check sites and surveillance of mf prevalence in these sites was done strictly following WHO guidelines [10].

The region of Vatovavy Fitovinany, in southeastern Madagascar, has historically presented high levels of LF transmission [7,8]. In this region, the districts of Manakara Atsimo, Mananjary and Vohipeno had some of the highest CFA prevalence levels in the country (>40%, ICT) during the 2004 mapping survey conducted at baseline of the National Programme (Table 1). As a result, they were among the first districts to be targeted for MDA in 2006, and by 2016 had received 10 annual rounds of treatment. Adjacent to this area, the district of Ifanadiana, with a baseline mf prevalence estimated at 31% from an earlier study [13], started MDA one year later, in 2007. Coverage in all four districts has been consistently above 65% for the whole period, although MDA campaigns did not happen at exact one year intervals (Fig 2). Moreover, periodic surveillance in sentinel and spot-check sites revealed that, while all four districts have experienced dramatic prevalence reductions since the beginning of the programme, only Manakara Atsimo, Mananjary and Vohipeno had achieved levels of microfilaraemia below 1% (Fig 2).

**Table 1:** Lymphatic filariasis baseline characteristics for each health district before initiation of MDA.

	Manakara Atsimo	Mananjary	Vohipeno	Ifanadiana^1^
District population	417,433	285,543	104,804	163,321
**National LF mapping**				
Population sampled	100	100	150	-
ICT positive (%)^3^	46 (46.0%)	58 (58.0%)	66 (44.0%)	-
**Sentinel site**^2^				
Population sampled	512	538	578	673
mf positive (%)	128 (25.0%)	107 (19.9%)	142 (24.6%)	98 (14.6%)

^1^ Ifanadiana was excluded from the 2004 mapping, since a mf prevalence of 31% had been estimated during an earlier survey by Champetier *et al*. (1996)

^2^ Baseline for Ifanadiana sentinel site was early 2007 instead of late 2005, since this district started MDA one year later.

^3^ LF mapping was done in 2004, and the ICT was used to assess LF CFA prevalence.

**Fig 3:**
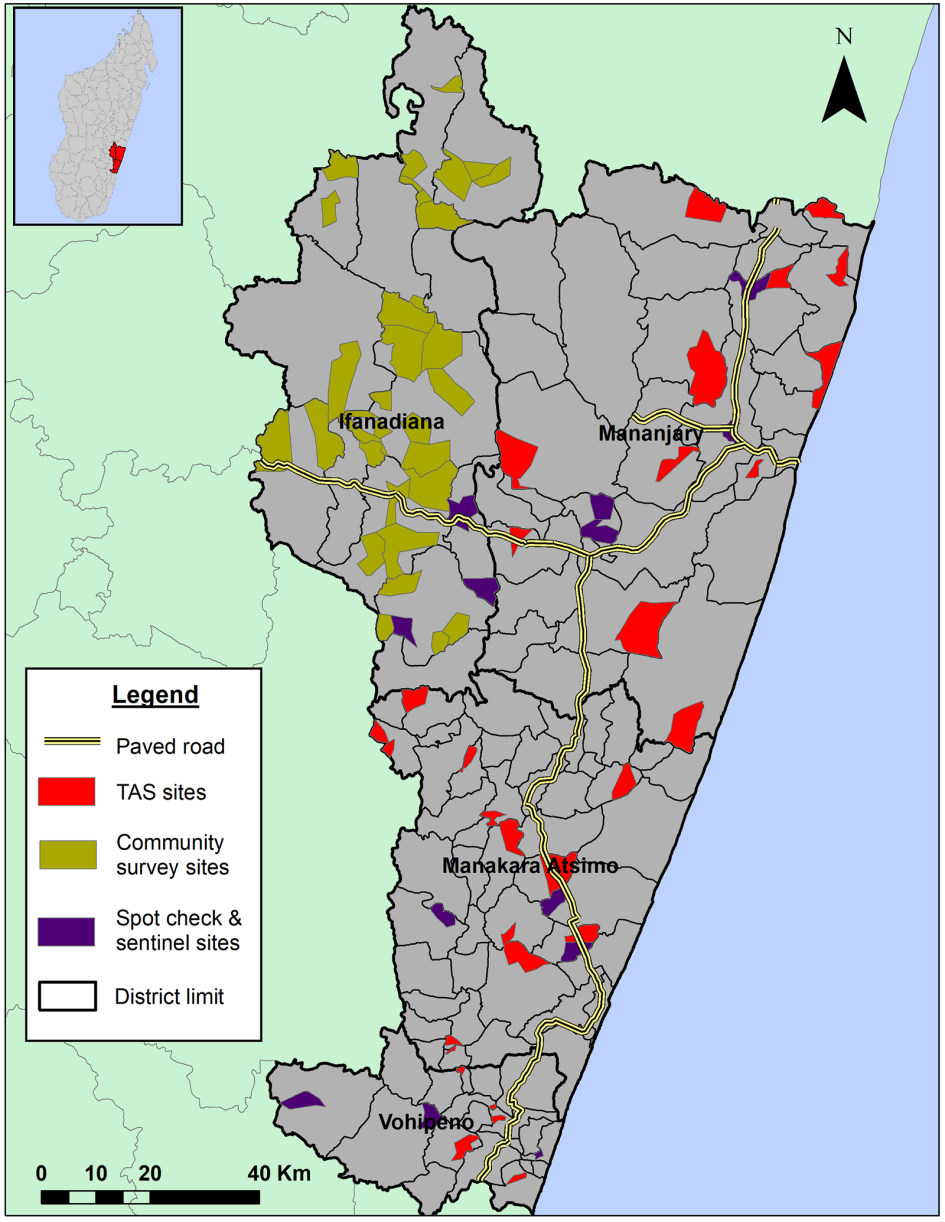
Sampling locations of the three different lymphatic filariasis prevalence surveys that took place in the region of Vatovavy Fitovinany, November-December 2016. Map was created by the authors with ArcMap software, version 10.4.1 using administrative layers from the Government of Madagascar and GPS information from the prevalence surveys.

This present study included three surveys carried out between November and December 2016 by separate teams in coordination with the National Programme (Fig 3). These include i) a school-based TAS carried out in the districts of Manakara, Mananjary, and Vohipeno (grouped as one evaluation unit, referred hereafter as “MAMAVO”); ii) a community survey in the adjacent district of Ifanadiana to obtain district-representative prevalence estimates; and iii) surveys in sentinel and spot-check sites of these four districts as part of the routine surveillance activities in the region. The two latter surveys were community-based and were targeted at children >5 years and adults of all ages. Despite the different designs, all surveys used the same diagnostic tests (FTS), and all teams received training and support from the National Programme for field survey execution.

### Transmission assessment survey (TAS)

The districts of Manakara, Mananjary and Vohipeno were merged into one single evaluation unit (EU “MAMAVO”) for the TAS evaluation, given their similarities in epidemiology and programme coverage over the past ten years (Fig 1). Survey design and execution was done strictly following WHO guidelines [10], with the support of “Survey Sample Builder” software (SSB, developed by the NTDs Support Centre) for sample size estimation and randomization. A school-based cluster survey was selected, with a total sample size of 1,692 children divided in 30 randomly selected schools. Detailed information on selected and replacement schools is available in S1 Table.

Before the beginning of the survey, authorities from the Ministry of Health and the Ministry of Education at the national, regional and district level participated in a three-day session of training and microplanning. School directors were informed of the survey in advance, and consent was obtained from the parents for inclusion of their children. Fieldwork was conducted between November 11–29 2016 by three teams of three people, including a lab technician, a district representative from the ministry of health, and a team member from the National Programme. At each school, all targeted children were organized in lines and were selected according to pre-existing lists produced by SSB, assisted by school teachers and staff. The TAS was continued until every randomly selected children in the target grades of all thirty schools had been tested. Thus, final sample size was larger than recommended (N = 1,825) due to differences in expected and actual class sizes and non-response rates.

### Community prevalence survey

A community survey to obtain district-representative estimates of FTS prevalence was conducted only in the district of Ifanadiana, which was not yet eligible for a TAS in 2016. To take into account spatial heterogeneity of LF, 545 individuals of ages 5 years or older were sampled using a cluster sampling scheme, with 30 random clusters at the fokontany level (smallest administrative unit consisting of several villages). Since villages in Ifanadiana vary greatly in terms of population size, one target village was selected and two additional villages from the same fokontany were used for replacement in case of insufficient sample sizes. Enumeration and selection of a random sample of 18 households within each village was done upon arrival by the field teams, in collaboration with the head of the village and community health workers. One member of each selected household was selected at random, excluding children under 5 years of age. Written consent was obtained from all survey participants, or their parents in the case of children. Fieldwork was coordinated by the National Institute of Public and Community Health (INSPC) and conducted between November 11–30 2016 by three teams of three people, each including a medical doctor, a lab technician, and a paramedic.

Besides the FTS prevalence rate, the survey included a brief questionnaire to gain a better understanding of the reasons why Ifanadiana was falling behind at LF elimination. Information in the questionnaire included demographic and socio-economic characteristics, knowledge related to MDA strategies, and previous practices to prevent LF such as preventive chemotherapy uptake and bed-net use.

### Community survey in sentinel and spot-check sites

As part of the LF monitoring and evaluation framework, WHO recommends National Programmes to routinely assess LF prevalence in both sentinel and spot-check sites. Sentinel sites are villages with a stable population of at least 500 inhabitants in an area of known high transmission or where achieving high coverage is difficult, and they are consistently surveyed since the beginning of the programme [10]. Spot-check sites have the same characteristics as sentinel sites, but are randomly chosen every year to avoid potential biases linked to program execution in sentinel sites. Selection of sites by the National Programme is done in collaboration with the district medical inspector, who provides a tentative list of villages that meet the above criteria. A lack of information about vector abundance or parasite prevalence means that site selection is based on symptomatic (chronic) LF cases known by the district officials or on locations where low MDA coverage rates have been reported. Following site selection, a convenience sample of 300 people is done, whereby the population is informed that LF testing will be carried out and volunteers meet the team in order to be tested.

As part of a World Bank project [14] that provided financial support for the fight against multiple NTDs in southeastern Madagascar, a prevalence survey was carried out in sentinel and spot-check sites of MAMAVO and Ifanadiana (among others) in 2016 to obtain updated prevalence estimates for the routine LF surveillance strategy of the National Programme. The sentinel site of each health district was included (same site as in previous surveys). Two spot-check sites per district (only one in Ifanadiana) were selected at random among villages of at least 500 inhabitants that were located further than 5 km from a health center. At each site, a convenience sample of 300 people was done among individuals of ages 5 years or older who agreed to participate in the study. While previous surveys in sentinel and spot-check sites had only assessed microfilaraemia, here CFA infection status of individuals was tested by FTS first, and then microfilaraemia was assessed for FTS positive cases. Similar to the community survey in Ifanadiana, this survey included a brief Knowledge, Attitudes and Practices (KAP) questionnaire administered to all participants. Fieldwork was coordinated by the National Malaria Control Program and conducted between November 23 and December 24 2016 by 4 teams of 3 people, each including a medical doctor, a lab technician or a paramedic, and 2 laboratory technicians.

### Assessment of infection status by CFA and microfilaraemia

In all three surveys, CFA was detected using FTS according to the manufacturer’s instructions. Briefly, 75μL of finger stick blood were collected between 8am and 5pm using the supplied micropipette, and the blood sample was added to the sample pad of the strip. Test results were read after 10 minutes, and any invalid tests (e.g. lack of a visible negative control line) were repeated. In addition, in the survey conducted at sentinel and spot-check sites, all positive FTS were examined for microfilaraemia. For these, a second sample of capillary blood was taken between 10pm-1am and was spread on a thin blood smear to evaluate the presence of microfilaraemia by microscopy.

### Statistical analyses

For each survey, demographic characteristics of the study population were summarized and FTS prevalence rates were estimated for each age group and district. Levels of knowledge, attitudes and practices related to LF transmission were estimated for the overall population and for each age group. Differences between age groups were assessed using Pearson chi-square tests (two sided), and p-values were reported. Associations of FTS positivity with knowledge, attitudes and practices were studied. Odds ratios and p-values of these associations were estimated through univariate conditional logistic regressions, using the age group as matching variable. All data analyses were carried out using R software, version 3.4.1 and R package “survival”, version 2.41–3. Figures were created with R package “ggplot2”, version 2.2.1. Maps were developed using ArcMap, version 10.4.1.

### Ethics statement

Written consent was requested directly from all individuals (or their parents in the case of children) and obtained prior to participation in the community-based surveys. For the TAS, the national programme coordinated with the school directors to inform the parents in advance of the survey and seek permission to include their children in case they were selected via a written note. Permission for their children’s participation was written on the parent-teacher contact book and brought by the child to the school. All three surveys received the approval of the Madagascar National Ethics Committee. Individuals who tested positive were treated with a combination of albendazole and diethylcarbamazine, and those who presented signs of chronic infections were advised to seek treatment at the nearest health center. All statistical analyses were carried out on de-identified data in order to protect individuals’ confidentiality.

## Results

### Demographic characteristics of survey participants

A total of 1,825 children participated in the TAS conducted in MAMAVO, 545 individuals of all ages participated in the Ifanadiana survey, and 3,325 individuals participated in sentinel and spot-check surveys in the four districts (Table 2). Most of the children included in the TAS (85.7%) had ages ranging between 5–7 years. Since the survey was targeted at first and second year classes at primary schools, rather than strict age ranges, some older children were also sampled (261 children ages 8–14 years). For sentinel and spot-check sites, children of ages 5–14 years represented about 35–50% of the population sampled. This proportion was significantly lower in the cluster survey at Ifanadiana, where they represented 20% of the sample. The sex ratio in the different surveys was balanced, although males made up for a smaller share in the sentinel and spot-check surveys (sex ratio 0.81). Information by district is available in S2 Table.

**Table 2:** Demographic characteristics of study participants at each of the three surveys.

	TAS	Sentinel & Spot-check	Community survey
**Sex**			
Male	930 (0.51)	1,473 (0.44)	277 (0.51)
Female	895 (0.49)	1,819 (0.55)	268 (0.49)
Sex Ratio	1.03	0.81	1.03
**Age group**			
5–7	1,563 (0.85)	379 (0.11)	26 (0.05)
8–14	261 (0.14)^1^	927 (0.28)	84 (0.15)
15–45	0	1,578 (0.48)	309 (0.57)
46–90	0	396 (0.12)	126 (0.23)
**Total**	1,825	3,325	545

^1^The survey was targeted at first and second year classes at primary schools, thus some children older than 7 were sampled

### Antigenaemia and parasitological results

None of the 1,825 school-enrolled children that were sampled in the TAS tested positive to FTS (Table 3). This was consistent with results from sentinel and spot-check sites, which tested an additional 1,306 children of ages 5–14 years. None of the children in the 5–7 age group, and only one child in the age range 8–14 years in these routine surveys tested positive. Despite consistent negative results for children in these three districts, prevalence in adults was still high in two of them. Ten out of 431 people aged 15–45 (2.3%) and 9 out of 110 people aged 46–90 (8.2%) tested positive in Mananjary District. For Vohipeno, prevalence was 8.5% in ages 15–45 and 13.3% in ages 46–90, out of 421 and 135 people tested respectively. Two out of 79 adults in Mananjary and Vohipeno who tested positive to FTS had also detectable levels of microfilaria in their blood. Results for the adjacent district of Ifanadiana varied significantly depending on the survey. Results from sentinel and spot-check sites suggested that prevalence ranged from 1.5–2.0% in adults (ages 15–45), while all children under 15 years tested negative. In contrast, prevalence estimates from the randomized survey conducted in 30 clusters across the district were consistently higher for all age groups. Prevalence in this survey was below 4% for children under 15 years, increasing progressively with age to 15.5% in the 15–45 age group (95% CI 11.8–20.2) and up to 27.8% in adults aged 46–90 years (95% CI 20.4–36.6). Geographical distribution of positive cases was heterogeneous across Ifanadiana, with prevalence ranging from zero (in six of the fokontany) up to 30–40% (in five other fokontany), although sample sizes were too small and did not allow for robust estimations at the cluster level.

**Table 3:** Prevalence of lymphatic filariasis (%) in the region of Vatovavy Fitovinany in 2016, assessed by Filariasis Test Strips.

	Manakara Atsimo		Mananjary		Vohipeno		Ifanadiana	
Age group	TAS	Sentinel & Spot-check	TAS	Sentinel & Spot-check	TAS	Sentinel & Spot-check	Community survey	Sentinel & Spot-check
5–7	0	0	0	0	0	0	3.85 (0.2–21.58)	0
8–14	0	0	0	0.41 (0.02–2.64)	0	0	2.38 (0.41–9.14)	0
15–45	-	0	-	2.32 (1.18–4.37)	-	8.55 (6.14–11.75)^1^	15.53 (11.78–20.17)	1.49 (0.48–4.04)
46–90	-	0	-	8.18 (4.05–15.38)^1^	-	13.33 (8.31–20.52)	27.78 (20.35–36.58)	1.89 (0.1–11.38)
Total	0	0	0	2.34 (1.49–3.61)	0	6.04 (4.62–7.85)	15.78 (12.88–19.18)	0.81 (0.3–1.99)

^1^ One mf positive case out of all FTS positive cases in Sentinel & Spot-Check sites was detected in each of these two populations

### Knowledge, attitudes and practices (KAP) related to LF transmission

The KAP survey conducted at sentinel and spot-check sites in the four districts under study revealed that only 51.2% of the population surveyed reported taking MDA in the previous year, 63.8% in the previous 5 years (Table 4). Individuals aged 5–14 years reported lower uptake rates (45.6%) than those aged 15–90 years (54.8%). In contrast to the low MDA uptake rates, both bed net ownership and use were high in all age groups. More than 90% of people reported owning or sleeping under a bed net, and 88.2% reported sleeping under a bed net every day in the past year. Knowledge about LF was low in this population (42.2%) and varied significantly among age groups (Table 4). Less than one in ten individuals aged 5–14 years reported knowing about LF, compared with nearly two thirds of those aged 15–90 years.

**Table 4:** Knowledge, attitudes and practices (KAP) related to LF transmission in sentinel and spot-check sites of Manakara Atsimo, Mananjary, Vohipeno and Ifanadiana districts (N = 3,325).

	Pop. mean (95% CI)	5–14 year mean (95% CI)	15–90 year mean (95% CI)	Chi2 p-value
**Preventive behaviours**				
Has taken MDA in the last 5 years	63.8 (62.1–65.5)	50.4 (47.5–53.4)	71.7 (69.7–73.7)	< 0.001
Has taken MDA in the last 12 months	51.2 (49.4–52.9)	45.6 (42.7–48.4)	54.8 (52.6–57.1)	< 0.001
Owns bednet	93.4 (92.5–94.2)	93.9 (92.6–95.2)	93.0 (91.9–94.1)	0.377
Sleeps under bednet	91.6 (90.7–92.6)	92.6 (91.2–94)	91.1 (89.9–92.4)	0.1563
Sleeps everyday under bednet (last 12 months)	88.2 (87.1–89.3)	89.1 (87.4–90.8)	87.7 (86.2–89.1)	0.2489
**Knowledge**				
Has attended 1^ary^ school or higher	85.4 (84.1–86.5)	91.9 (90.3–93.3)	81.3 (79.5–83)	<0.001
Knows about LF	42.2 (40.5–43.9)	8.9 (7.3–10.4)	63.7 (61.6–65.9)	< 0.001
- Information source				
Health staff	7.1 (6.3–8)	1.6 (0.9–2.2)	10.8 (9.5–12.2)	< 0.001
Community health worker	18 (16.7–19.3)	3.3 (2.4–4.3)	27.7 (25.7–29.6)	< 0.001
Other people	27.5 (26–29.1)	5.9 (4.6–7.2)	41.5 (39.3–43.6)	< 0.001
Radio	2.1 (1.6–2.5)	0.6 (0.2–1.1)	3 (2.2–3.7)	< 0.001
Newspaper^1^	0.2 (0.1–0.5)	0.1 (0–0.5)	0.3 (0.1–0.7)	-
Television^1^	0.3 (0.2–0.6)	0.1 (0–0.5)	0.5 (0.2–0.9)	-
- Knows about LF MDA	38 (36.3–39.7)	7.9 (6.4–9.3)	57.7 (55.5–59.9)	< 0.001

^1^ Insufficient variability and sample size to allow for appropriate estimations of Chi^2^ test

Individuals who reported taking MDA in the past 5 years had significantly lower odds of infection (Table 5), as assessed through FTS (OR = 0.57; 95% CI 0.36–0.91), and this protective effect was stronger for individuals who reported taking MDA in the past 12 months (OR = 0.2; 95% CI 0.11–0.35). Moreover, individuals who had attended primary school or higher had significantly lower odds of infection than those who had never attended school (OR = 0.5; 95% CI 0.3–0.81). In contrast, knowledge about LF or bed net use was not significantly associated with FTS positivity. Despite higher FTS prevalence levels found in Ifanadiana district-representative survey, this population reported higher MDA uptake rates (66.2%) and knowledge about MDA (68.4%), although bed net use was lower (S3 Table). In this survey, MDA uptake and knowledge were not associated with FTS positivity (S4 Table). Only attendance to primary school or higher was significantly associated with a lower odds of infection (OR = 0.55; 95% CI 0.32–0.95).

**Table 5:** Factors associated with FTS positivity in sentinel and spot-check sites of Manakara, Mananjary, Vohipeno and Ifanadiana^1^ (N = 3,325).

	Mean in FTS- (%)	Mean in FTS+ (%)	Odds Ratio	95% CI	p-value
**Preventive behaviours**					
Has taken MDA in the last 5 years	63.9	58.8	0.57	(0.36–0.91)	0.0175
Has taken MDA in the last 12 months	52.0	20.0	0.2	(0.11–0.35)	<0.0001
Owns bed net	93.5	90.0	0.65	(0.31–1.39)	0.2671
Sleeps under bed net	91.7	88.8	0.86	(0.41–1.81)	0.6828
Sleeps everyday under bed net (last 12 months)	88.3	85	0.86	(0.45–1.65)	0.6562
**Knowledge**					
Has attended 1^ary^ school or higher	85.7	69.6	0.5	(0.3–0.81)	0.0056
Knows about LF	41.8	58.8	0.81	(0.51–1.28)	0.363
- Information source					
Health staff	7.1	7.5	0.68	(0.29–1.57)	0.3628
Community health worker	17.7	30	1.17	(0.71–1.91)	0.539
Other people	27.3	36.2	0.78	(0.49–1.25)	0.3047
Radio	2.1	1.2	0.42	(0.06–3.04)	0.3879
Newspaper^2^	0.2	0	-	-	-
Television^2^	0.3	0	-	-	-
- Knows about LF MDA	37.7	49.4	0.7	(0.45–1.11)	0.1276

^1^ Univariate conditional logistic regressions. All regressions were matched by age group (5–14 vs 15–90 years)

^2^ Insufficient variability and sample size to allow for appropriate estimations of Odds Ratio and p-value

## Discussion

At the turn of the 20th century, it was estimated that 120 million people were infected with LF globally and more than one billion were at risk of infection [3]. A strategy of MDA, following the 1997 World Health Assembly resolution to eliminate LF, has led to one of the most ambitious and successful interventions against a neglected tropical disease. Under sufficient levels of intervention coverage, transmission of LF can be interrupted within five years. To allow comparable monitoring and evaluation of national programmes, WHO designed an exhaustive plan that involves routine surveillance and the implementation of TAS to identify the areas where MDA can be stopped. Here we report on the results of the first TAS conducted in Madagascar (after the small district-island of Saint Marie), together with two other complementary prevalence surveys. We show that TAS results confirmed the consistent decline in LF observed in recent years and suggest that MDA can be stopped in our study area. However, the high FTS prevalence levels observed in the adult population, together with low reported MDA uptake rates, raise questions about a potential risk for recrudescence (e.g. if adult worms present in these populations are eventually able to produce microfilariae).

After ten rounds of MDA, we found no FTS positive children during the TAS conducted in three districts of southeastern Madagascar. This represents the first evidence for Madagascar that transmission is being halted in traditionally high endemic districts, and that MDA may be partially stopped in the country. These results are consistent with those reported in other endemic countries of Africa and Asia, where scale-up of TAS has allowed confirmation in multiple regions of a reduction in LF transmission below sustainable levels [15–18]. Despite progress, Madagascar still faces considerable challenges for LF elimination and its evaluation. Political instability together with a sudden drop in official development assistance between 2009 and 2014 contributed to the interruption of routine surveillance efforts in this period. Furthermore, a TAS scale-up has not recently been possible in the country due to financial constraints (a TAS has a median cost of over $20,000 [19]), despite having many districts with 5–10 rounds of MDA that fulfill the conditions for such evaluation. As a result, the National Programme continues to distribute preventive chemotherapy in potentially non-endemic districts, using resources that could be allocated to expanding the geographical reach of the MDA strategy, achieving nation-wide coverage.

The different surveys we conducted simultaneously in the same geographical area provided seemingly contradictory results and illustrate the complexity of evaluating lymphatic filariasis transmission. First, 2016 surveys in sentinel and spot-check sites of Mananjary and Vohipeno, with CFA prevalence levels over 2%, suggest that in retrospect a TAS should have not been conducted in these districts. However, the National Programme followed WHO guidelines regarding TAS eligibility [10] and the most recent surveys from 2013 had estimated levels of microfilaraemia lower than 1%, which was still the case in 2016. Second, prevalence estimates obtained from the district-representative survey in Ifanadiana were nearly twenty times higher than those from sentinel and spot-check sites in the district. It is worth noting that results were not adjusted for unequal probability of selection among individuals due to a lack of robust population estimates, which could have led to biases in the point estimates and confidence intervals. Yet, the gap is staggering and suggests that sentinel and spot-check sites were not representative of the district as a whole. WHO guidelines recommend that programmes select sites of known high transmission or low MDA coverage [10]. However, in low-resource settings where information about vectors or parasite prevalence is scarce, selection is frequently based on symptomatic LF cases, which do not necessarily reflect current transmission. In addition, the limited capacity of the National Programme to supervise community distribution of MDA could lead to inadequate administration (e.g. not directly observed uptake) or reporting, thus biasing coverage estimates and further undermining selection criteria for surveillance in sentinel and spot-check sites.

The high prevalence of antigenaemia observed in adults of three out of the four districts evaluated, after nine years or more of MDA, suggests that these populations are still infected with adult worms. A reason for including only children in TAS evaluations is that some treatments against LF (including diethylcarbamazine) have both partially macrofilaricidal and sterilizing effects on adult worms [20–23]. Thus, transmission may be stopped even when worms are still temporarily present in the population. However, the effectiveness and duration of these sterilizing effects are poorly understood. The presence of an infected pool of adults in the population could not only constitute a potential source of new infections, but can also increase the susceptibility for childhood infections mediated by in-utero immunological mechanisms [24,25]. Given the low number of microfilaremic individuals (2 out of 79 FTS positive in sentinel and spot-check sites), an immediate recrudescence of LF cases is unlikely. Yet, the longevity of the adult worms (5–7 years or more) coupled with high FTS prevalence, could indicate a risk for a recrudescence after a few years if MDA is stopped. This risk could be amplified by the nearby populations of Ifanadiana, where FTS district-prevalence was higher than 15%. The presence of such “hotspots” is consistent with previous studies in other African countries where some EUs have remained endemic even after 14 rounds of MDA, despite overall progress in nearby regions [26].

The results observed in our study, radically different for each age group, could be the result of distinct strategies implemented at schools and communities. A key component of the Madagascar MDA strategy is the school-based distribution of chemotherapy for all enrolled children 5–14 years of age, in partnership with other NTD programmes. For all other children and adults, MDA is facilitated through community health workers who distribute treatments on a door-to-door basis. However, uptake during community administration by health workers can be considerably more challenging, since household members may be absent, adults can decide not to take the drugs, or health workers may not be able to cover their assigned area [27]. Indeed, many studies have found very low uptake among adult populations (around 50%) due to a variety of context-specific factors [27–29]. Although results from KAP surveys of sentinel and spot-check sites did not support this hypothesis, the low level of knowledge about LF found in these children (less than one in ten) could have biased their responses. Since WHO guidelines promote an “integrated package” of NTD interventions that target children at schools [30–32], further studies should consider its potential impact on TAS results. Indeed, the current number of teachers participating in administration of preventive chemotherapy for NTDs globally is almost twice the number of health workers [30].

In conclusion, our study presents the recent progress in eliminating LF in Madagascar with the first successful TAS results in the island, but it suggests that evaluations of LF transmission may in some epidemiological contexts be more complex than generally recognized in international guidelines. Logistical local problems as well as ecological reasons may explain the heterogeneity of the results for different settings. In contexts where MDA for LF includes school-based administration, it may be appropriate to conduct prevalence surveys for the adult population or children not enrolled in school to complement the information gained from TAS results. As shown for Ifanadiana, cluster randomization for these complementary surveys may be important in order to gain a full geographical picture of transmission.

## Supporting information

S1 Table List of selected schools for the TAS survey, and replacement schools sorted by order of replacement. (DOCX)

S2 Table Demographic characteristics of study participants at each survey, by health district. (DOCX)

S3 Table Knowledge, attitudes and practices (KAP) related to LF transmission in communities of Ifanadiana district-representative survey. (DOCX)

S4 Table Factors associated with FTS positivity in Ifanadiana district-representative survey. (DOCX)

S1 Dataset It contains all de-identified data used in this study. The data for each of the three surveys is presented in a separate excel sheet.
(XLSX)
