# Supplementary material for: Towards elimination of lymphatic filariasis in southeastern Madagascar: Successes and challenges for interrupting transmission
Source: PLoS Negl Trop Dis. 2018 Sep 17;12(9):e0006780. doi: 10.1371/journal.pntd.0006780 (PMC6160210; doi:10.1371/journal.pntd.0006780)
Supplement: S1 Table — (DOCX) [file pntd.0006780.s001.docx]

**S1 Table.** List of selected schools for the TAS survey, and replacement schools sorted by order of replacement

| **DISTRICT** | **COMMUNE** | | **FOKONTANY** | | | | **SCHOOL** | | |
| --- | --- | --- | --- | --- | --- | --- | --- | --- | --- |
| **SELECTED SCHOOLS** | | |  | | | |  | | |
| MANANJARY | MAROFOTOTRA | | AMBOHITSARA I | | EPP AMBOHITSARA I | | |  |  |
|  | MAHELA | | AMBALAHADY | | EPP ANTAVIRANAMBO | | |  |  |
|  | MAROSANGY | | ANDRORANGA | | EPP ANDRORANGA | | |  |  |
|  | MORAFENO | | MORAFENO | | ECOLE COMMUNAUTAIRE AMBOHIMIARINA | | |  |  |
|  | MAHATSARA-ATSIMO | | AMBOANATO | | EPP AMPANDIMANA | | |  |  |
|  | AMBOHINIHAONANA | | AMBODIMANGA NOSIVOLO | | EPP AMBODIMANGA NOSIVOLO | | |  |  |
|  | MAROKARIMA | | MAROKARIMA | | EPP ANKEBA | | |  |  |
|  | TSARAVARY | | TANAMBAO - MAROFODY | | EPP TANAMBAO MAROFODY | | |  |  |
|  | KIANJAVATO | | FOTOBOHITRA | | EPP FOTOBOHITRA | | |  |  |
|  | ANOSIMPARIHY | | AMBALAMAINTY | | EPP SAHAKONDRO | | |  |  |
|  | NAMORONA | | VALANADY | | ECOLE COMMUNAUTAIRE ANTSAHALAVA | | |  |  |
|  | AMBOHITSARA-EST | | AMBODIPAISO | | EPP AMBODIROTRA | | |  |  |
| MANAKARA | SAHAREFO | | AMBOAIFITO | | EPP AMBOAIFITO | | |  |  |
|  | ONILAHY | | ONILAHY | | EPP AMBOHIMAHATSINJO | | |  |  |
|  | FENOMBY | | MANAKANA | | EPP MANAKANA | | |  |  |
|  | AMBOTAKA | | AMBOTAKA | | EPP ANDRANOVATO | | |  |  |
|  | SAHASINAKA | | VANGAINDRANOKELY | | ECOLE COMMUNAUTAIRE VOHIBOLA | | |  |  |
|  | AMBOHITSARA\"M\" | | KIANJANOMBY | | ECOLE COMMUNAUTAIRE MAHAVANONA | | |  |  |
|  | MIZILO GARE | | MIZILO GARE | | EPP AMBALATENINA MIZILO | | |  |  |
|  | AMBOANJO | | VOHITRAVOHA | | EPP VOHITRAVOHA | | |  |  |
|  | MAROFARIHY | | MAROFARIHY | | EPP MAROFARIHY | | |  |  |
|  | MANJARIVO | | ANKARIMALAZA | | EPP AMBOHITSARA | | |  |  |
|  | MANAKARA | | TANAMBAO OMBIMENA | | ECOLE PRIVEE LUTHERIENNE | | |  |  |
|  | LOKOMBY | | MITANTY EST | | EPP MITANTY EST | | |  |  |
|  | VATANA | | FOTATRA | | EPP FOTATRA | | |  |  |
| VOHIPENO | MAHASOABE | | ANDRANOTSARA | | COLLEGE ISLAMIQUE D\'ENSEIGNEMENT GENERAL ANDRANOTSARA | | |  |  |
|  | ANKARIMBARY | | MAROAKANJO | | ECOLE PRIVEE AL-HIDAYIAT DE MAROAKANJO | | |  |  |
|  | VOHIPENO | | VATOLAPANA | | EPP VATOLAPANA | | |  |  |
|  | IFATSY | | KARIMBELO Z | | EPP AMBOANGIBE | | |  |  |
|  | VOHINDAVA | | NOHONA | | EPP NOHONA | | |  |  |
| **REPLACEMENT SCHOOLS** | | | |  | |  | | |  |
| MANAKARA | | AMBALAVERO | | AMBODILAZA | | EPP AMBODILAZA | | |  |
| VOHIPENO | | MAHASOABE | | MAROMBY | | EPP TSARAMANDROSO | | |  |
| MANANJARY | | MAHELA | | AMBODIMANGA II | | EPP VOHIMASINA | | |  |
| VOHIPENO | | IFATSY | | IFATSY | | EPP IFATSY | | |  |
| MANAKARA | | MANAKARA | | MAROALAKELY | | ECOLE PRIVEE LES COCCINELLES | | |  |
| MANANJARY | | MAHATSARA-ATSIMO | | IAMBORANO | | EPP AMBALAHADY | | |  |
| MANANJARY | | ANKATAFANA | | AMPANGALANA SUD | | EPP AMPANGALANA SUD | | |  |
| MANAKARA | | ANOROMBATO | | ANALAVORY | | EPP ANALAVORY | | |  |
| MANANJARY | | NAMORONA | | MAHASOA | | EPP MAHATSARA | | |  |
| MANAKARA | | MAHAMAIBE | | ANOROMBATO | | EPP AMBOHIMANDROSO ANOROMBATO | | |  |
