# Supplementary material for: Towards elimination of lymphatic filariasis in southeastern Madagascar: Successes and challenges for interrupting transmission
Source: PLoS Negl Trop Dis. 2018 Sep 17;12(9):e0006780. doi: 10.1371/journal.pntd.0006780 (PMC6160210; doi:10.1371/journal.pntd.0006780)
Supplement: S2 Table — (DOCX) [file pntd.0006780.s002.docx]

**S2 Table.** Demographic characteristics of study participants at each survey, by health district

|  | **MANAKARA-ATSIMO** | | **MANANJARY** | | **VOHIPENO** | | **IFANADIANA** | |
| --- | --- | --- | --- | --- | --- | --- | --- | --- |
|  | **TAS** | **Sentinel & Spot-check** | **TAS** | **Sentinel & Spot-check** | **TAS** | **Sentinel & Spot-check** | **Community survey** | **Sentinel & Spot-check** |
| **Sex** |  |  |  |  |  |  |  |  |
| Male | 485 (0.49) | 416 (0.47) | 317 (0.52) | 387 (0.43) | 128 (0.56) | 396 (0.44) | 277 (0.51) | 274 (0.45) |
| Female | 506 (0.51) | 471 (0.53) | 288 (0.48) | 509 (0.57) | 101 (0.44) | 501 (0.56) | 268 (0.49) | 338 (0.55) |
| Sex Ratio | 0,96 | 0,88 | 1,10 | 0,76 | 1,27 | 0,79 | 1,03 | 0,81 |
| **Age group** |  |  |  |  |  |  |  |  |
| 5-7 | 771 (0.78) | 103 (0.12) | 584 (0.97) | 111 (0.12) | 208 (0.91) | 98 (0.11) | 26 (0.05) | 67 (0.11) |
| 8-14 | 220 (0.22) | 219 (0.25) | 20 (0.03) | 242 (0.27) | 21 (0.09) | 238 (0.27) | 84 (0.15) | 228 (0.37) |
| 15-45 | - | 458 (0.52) | - | 431 (0.48) | - | 421 (0.47) | 309 (0.57) | 268 (0.44) |
| 46-90 | - | 98 (0.11) | - | 110 (0.12) | - | 135 (0.15) | 126 (0.23) | 53 (0.09) |
| **Total** | 991 | 898 | 605 | 899 | 229 | 910 | 545 | 618 |
