# Supplementary material for: Towards elimination of lymphatic filariasis in southeastern Madagascar: Successes and challenges for interrupting transmission
Source: PLoS Negl Trop Dis. 2018 Sep 17;12(9):e0006780. doi: 10.1371/journal.pntd.0006780 (PMC6160210; doi:10.1371/journal.pntd.0006780)
Supplement: S3 Table — (DOCX) [file pntd.0006780.s003.docx]

**S3 Table.** Knowledge, attitudes and practices (KAP) related to LF transmission in communities of Ifanadiana district-representative survey (N=545)

|  | **Pop. mean**  **(95% CI)** | **5-14 year mean**  **(95% CI)** | **15-90 year mean**  **(95% CI)** | **Chi2**  **p-value** |
| --- | --- | --- | --- | --- |
| **Preventive behaviours** |  |  |  |  |
| Has taken MDA during last round | 66.2 (62.1-70.2) | 72.7 (63.3-80.6) | 64.7 (59.9-69.1) | 0,1377 |
| Slept under a bed net the previous night | 82.9 (79.5-85.9) | 77.3 (68.1-84.5) | 84.5 (80.7-87.7) | 0,0952 |
| **Knowledge** |  |  |  |  |
| Has attended 1^ary^ school or higher | 82.6 (79.1-85.6) | 93.6 (86.9-97.2) | 79.7 (75.5-83.3) | 0,001 |
| Knows about MDA | 68.4 (64.3-72.3) | 70.9 (61.4-79) | 67.9 (63.2-72.2) | 0,6226 |
| - Knowledge source |  |  |  |  |
| Health staff | 5.5 (3.8-7.9) | 1.8 (0.3-7.1) | 6.5 (4.4-9.3) | 0,0946 |
| Community health worker | 53 (48.7-57.3) | 28.2 (20.2-37.7) | 59.4 (54.5-64) | < 0.001 |
| Friend or neighbour | 2.4 (1.3-4.2) | 0.9 (0-5.7) | 2.8 (1.5-4.9) | 0,4285 |
| Teacher | 9.7 (7.4-12.6) | 41.8 (32.6-51.6) | 1.6 (0.7-3.5) | < 0.001 |
| Newspaper or panflet^1^ | 0.2 (0-1.2) | 0 (0-4.2) | 0.2 (0-1.5) | - |
| Radio^1^ | 0.6 (0.1-1.7) | 0 (0-4.2) | 0.7 (0.2-2.2) | - |

^1^ Insufficient variability and sample size to allow for appropriate estimations of Chi2 test
