# Supplementary material for: Towards elimination of lymphatic filariasis in southeastern Madagascar: Successes and challenges for interrupting transmission
Source: PLoS Negl Trop Dis. 2018 Sep 17;12(9):e0006780. doi: 10.1371/journal.pntd.0006780 (PMC6160210; doi:10.1371/journal.pntd.0006780)
Supplement: S4 Table — (DOCX) [file pntd.0006780.s004.docx]

**S4 Table.** Factors associated with FTS positivity in Ifanadiana district-representative survey^1^ (N=545)

|  | **Mean in FTS- (%)** | **Mean in FTS+ (%)** | **Odds Ratio** | **95% CI** | **p-value** |
| --- | --- | --- | --- | --- | --- |
| **Preventive behaviours** |  |  |  |  |  |
| Has taken MDA during last round | 66.4 | 65.1 | 0.99 | 0.6-1.61 | 0.9584 |
| Slept under a bed net the previous night | 82.4 | 86 | 1.17 | 0.6-2.27 | 0.6523 |
| **Knowledge** |  |  |  |  |  |
| Has attended 1^ary^ school or higher | 84.5 | 72.1 | 0.55 | 0.32-0.95 | 0.0325 |
| Knows about MDA | 68.2 | 69.8 | 1.08 | 0.65-1.8 | 0.7585 |
| - Knowledge source |  |  |  |  |  |
| Health staff | 5.4 | 5.8 | 0.91 | 0.34-2.47 | 0.8598 |
| Community health worker | 51.6 | 60.5 | 1.09 | 0.68-1.77 | 0.7162 |
| Friend or neighbour | 2.2 | 3.5 | 1.42 | 0.38-5.31 | 0.6056 |
| Teacher | 11.1 | 2.3 | 0.7 | 0.14-3.48 | 0.6647 |
| Newspaper or panflet^2^ | 0.2 | 0 | - | - | - |
| Radio^2^ | 0.3 | 0 | - | - | - |

^1^ Univariate conditional logistic regressions. All regressions were matched by age group (5-14 vs 15-90)

^2^ Insufficient variability and sample size to allow for appropriate estimations of Odds Ratio and p-value
